# Variation in neonatal mortality and its relation to country characteristics in sub-Saharan Africa: an ecological study

**DOI:** 10.1136/bmjgh-2016-000209

**Published:** 2017-10-25

**Authors:** Gbenga Ayodele Kayode, Diederick E Grobbee, Mary Amoakoh-Coleman, Evelyn Ansah, Olalekan A Uthman, Kerstin Klipstein-Grobusch

**Affiliations:** 1 Julius Global Health, Julius Center for Health Sciences and Primary Care, University Medical Centre Utrecht, Utrecht, The Netherlands; 2 International Research Centre of Excellence, Institute of Human Virology, Abuja, Nigeria; 3 Global Geo and Health Data Center, Utrecht University, Utrecht, The Netherlands; 4 Postdoctoral Unit, Noguchi Memorial Institute for Medical Research, University of Ghana, Accra, Ghana; 5 Department of Epidemiology and Disease Control, School of Public Health, University of Ghana, Accra, Ghana; 6 Research and Development Division, Ghana Health Service, Accra, Ghana; 7 Warwick-Centre for Applied Health Research and Delivery, Division of Health Sciences, Warwick Medical School, The University of Warwick, Coventry, UK; 8 International Health Group, Liverpool School of Tropical Medicine, Liverpool, UK; 9 Division of Epidemiology and Biostatistics, Faculty of Health Science, School of Public Health, the University of Witwatersrand, Johannesburg, South Africa

**Keywords:** child health, health systems

## Abstract

**Background:**

A substantial reduction in neonatal mortality is the main priority to reduce under-five mortality. A clear understanding of the variation in neonatal mortality and the underlying causes is important for targeted intervention. We aimed to explore variation in neonatal mortality and identify underlying causes of variation in neonatal mortality in sub-Saharan Africa (SSA).

**Methods:**

This ecological study used 2012 publicly available data from WHO, the US Agency for International Development and the World Bank. Variation in neonatal mortality across 49 SSA countries was examined using control chart and explanatory spatial data analysis. Associations between country-level characteristics and neonatal mortality were examined using linear regression analysis.

**Results:**

The control chart showed that 28 (57%) SSA countries exhibited special-cause variation, 14 countries were below and 14 above the 99.8% control-limits. The remaining 21 (43%) SSA countries showed common-cause variation. No spatial clustering was observed for neonatal mortality (Global Moran’s I statistic −0.10; p=0.74). Linear regression analysis showed HIV/AIDS prevalence among the population of reproductive age to be positively associated with neonatal mortality (β=0.463; 95% CI 0.135 to 0.790; p<0.01). Declining socioeconomic deprivation (β=−0.234; 95% CI −0.424 to −0.044; p<0.05) and high quality of healthcare governance (β=−1.327, 95% CI −2.073 to −0.580; p<0.01) were inversely associated with neonatal mortality.

**Conclusion:**

This study shows a wide variation in neonatal mortality in SSA. A substantial part of this variation can be explained by differences in the quality of healthcare governance, prevalence of HIV and socioeconomic deprivation. Future studies should validate our findings using more rigorous epidemiological study designs.

**Key questions**

**What is already known about this topic?**

- Previous studies have linked variation in neonatal mortality with paternal, maternal, neonatal and socioeconomic factors at individual-level.
- In addition, experts in neonatal health have advocated for increase in health financing, healthcare coverage and other interventions as means of improving survival in early life.

**What are the new findings?**

- This study determined the impact of healthcare governance on neonatal survival, which no previous studies have examined.
- Increase in healthcare coverage and financing without good quality of healthcare governance may not improve neonatal survival.

**Recommendations for policy**

- As governments in SSA is increasing health financing, health human resources and health service coverage, it is important to improve the quality of healthcare governance to avoid wastage.

## Background

A substantial reduction in neonatal mortality is one of the main priorities to realise sustainable development goal 3 (SDG 3—aims to ensure healthy lives and promote well-being for all at all ages) given that neonatal death accounts for 40% of under-five mortality.^1^ Globally, about 10 000 neonates are dying daily,^2^ an estimate that may be significantly higher considering the likelihood of under-reporting,^3 4^ especially in low-income and middle-income countries.^5^ Sub-Saharan Africa (SSA) has the highest rate of neonatal mortality worldwide,^6^ with some degree of variation across SSA countries and only minor declines over the last two decades.^3^ Examining variation in health outcomes across similar settings is an important aspect of epidemiological research to guide assessment of inequality in health outcomes,^7 8^ health system performance monitoring^9–11^ and healthcare governance.^12^

Exploring variation in health outcome requires collection of valid data, appropriate application of statistical analyses and adequate knowledge of exploring variation. Several studies emphasised the approach proposed by Shewhart and Deming on how to investigate and deal with variation.^13–15^ According to their findings, variation is a product of multiple factors, and every system is subjected to both common (expected) and special-cause (unexpected) variation. This concept has been applied in health system research.^9 16^

In common-cause variation, it is expected that the deviation of the estimated health outcome for a particular setting should be within 3 standard deviations (SD) of the overall average of the estimated health outcome for all settings.^13^ In other words, the estimated health outcome should be within so-called ‘control limits’.^13^ Although such variation is referred to as common-cause variation, it does not imply that the causes of variation are identical across the settings within the control limits, but that a combination of different multiple factors might have influenced the observed health outcome in these settings. Thus, the combined effect of these multiple factors on health outcome for each population is similar, because the effect estimates are all within the control limits.

In case of special-cause variation, the health outcome of that setting is expected to be outside the 3 SD of the overall average of the estimated health outcomes for all the settings. Factors responsible for such variation usually have an unexpected high impact on the observed health outcome. To thoroughly examine variation in health outcome across different populations, revealing the existence of common-cause and special-cause variation is a key aspect. In addition, it is important to visualise spatial distribution of health outcomes across populations and settings to detect any unusual pattern in terms of spatial clustering and outliers. Findings from such investigations may provide evidence to formulate targeted interventions aimed to improve health outcomes.^17 18^

Beyond the assessment of variation in neonatal mortality across SSA, the current study seeks to identify underlying factors that could explain the variation in neonatal mortality by considering country characteristics such as quality of healthcare governance, health financing, human health resources, health service delivery and the country’s socioeconomic status. As SSA accommodates almost 70% of all people living with HIV globally^19^ with >500 000 newborns infected annually,^20^ we considered the prevalence of HIV among the population of reproductive age as a potential factor to explain variation in neonatal mortality.

## Methods 

### Study design and data collection 

This ecological study uses 2012 publicly available data from WHO,^21^ the US Agency for International Development^22^ and the World Bank^23^ repositories for 49 SSA countries. Aggregate data at country level on neonatal mortality, illiteracy and poverty rate, percentage of safe water coverage, improved sanitation facilities, HIV prevalence among the population of reproductive age (ie, 15–49 years), health governance, health human resources, health delivery and health financing were extracted.

### Study variables

#### Outcome (dependent)

Neonatal mortality was defined as the number of deaths within the first 28 days of life per 1000 live births.

#### Country-level characteristics (independent)

Country-level characteristics considered to explain the underlying causes of variation in neonatal mortality were:

1. Prevalence of HIV infection among the population of reproductive age (ie, 15–49 years); 
2. Health financing based on per capita total expenditure on health at purchasing power parity (PPP) measured in US$, that is, the ratio of total expenditure on health (private and public) and the total population; expressed in US$ per person per year;
3. Health service delivery assessed by the average proportion of the population using contraceptives among women aged 15–49 years and births attended by skilled health staff;
4. Health human resources assessed by the average number of physicians and nurses per 1000 people; 
5. Quality of health governance as assessed by political stability, government effectiveness, voice and accountability, rule of law, regulatory quality, political stability and control of corruption. Each of these six areas directly impacting health system operations was assessed based on points ranging from −2.5 to +2.5; higher points indicate better quality of health governance. Total health governance performance rating scores range from −15 to +15; higher scores indicate better quality of health governance, respectively. Detailed information on assessment of health governance has been published elsewhere.^24^
6. Country’s socioeconomic index was measured by use of the average proportion of the population that are literate, live above poverty and have access to safe water and improved sanitation facilities. Literacy rate was defined as the percentage of people aged 15 years and above who can read and write. Poverty rate was defined as the percentage of the population living below poverty line (<US$1.25/day).^22^

### Statistical analysis

#### Descriptive statistics

The range, mean and SD of neonatal mortality and country-level characteristics were estimated. A mixed-effect model was applied to estimate the weighted average of neonatal mortality using their variance as weight. Statistical analysis was performed using R statistical package.^25^

#### Control chart 

A control chart was constructed by plotting neonatal mortality rates on the y-axis against a measure of their precision, that is, SD on the x-axis. The chart has five horizontal lines, one central line with two lines below and above it. The central line indicates the overall neonatal mortality while the two additional lines at 95% limits (≈2 SD) and 99.8% limits (≈3 SD) on both sides of the central line represent 95% limits and 99.8% limits of the overall estimate of neonatal mortality. Countries with a neonatal mortality rate within the 99.8% control limits (≈3 SD) are considered to show common-cause variation while those outside the 99.8% control limits are considered to exhibit special-cause variation. Statistical analysis was performed using Stata statistical package V.11.^26^

#### Spatial data analysis

Pfeiffer *et al* and Anselin *et al* have described the application of Global Spatial Autocorrelation (GSA) and Local Indicators of Spatial Autocorrelation (LISA).^27 28^ In this study, we used Explanatory Spatial Data Analysis (ESDA) to estimate GSA that indicates overall clustering or non-randomness, that is, assessing the degree of overall similarity in the spatial pattern of neonatal mortality across SSA countries. Since GSA cannot express the degree of similarity between each country and its neighbouring countries with regard to neonatal mortality, we applied LISA to determine the presence of significant spatial patterns of neonatal mortality across SSA countries. Non-randomness in the spatial pattern of neonatal mortality was identified in terms of significant spatial clustering or outliers. The estimated Local Moran’s I statistic was used to assess the significance of local clusters and outliers. Overall, four identifiable categories of local spatial association or local spatial autocorrelation can be observed: two categories indicate clustering while the other two suggest outliers. Hot spot local spatial association (clustering of countries with high incidence of neonatal mortality) and cold spot local spatial association (clustering of countries with low incidence of neonatal mortality) indicate clustering. High-low local spatial association (a country with high incidence of neonatal mortality surrounded by neighbouring countries with low incidence of neonatal mortality) and low-high local spatial association (a country with low incidence of neonatal mortality surrounded by neighbouring countries with high incidence of neonatal mortality) indicate outliers.

#### Associations between country-level characteristics and neonatal mortality 

Associations between neonatal mortality and country-level characteristics were visualised by use of bar chart and two-way scatter plot with a prediction line and 95% CI. Pairwise correlation test was used to determine the strength of the relationships between country-level characteristics and neonatal mortality. Based on the observed Moran’s I statistic, spatial regression analysis was not considered^29^; instead a linear regression analysis was applied to examine the relationships between neonatal mortality and country-level characteristics. Univariable analysis was performed to examine the crude relationships between neonatal mortality and each country characteristic. Thereafter, all the prespecified country characteristics were included in a multivariable linear regression model. Model reduction was performed based on the significance of each variable and adjusted R^2^, country characteristics that were now statistically significant were removed one after the other and the model that explained the observed variation in neonatal mortality best was determined based on the adjusted R^2^ statistics of the model. The underlying assumptions for linear regression analysis were checked. We used visual scatter plot to check for linearity relationship between the predictors and neonatal deaths per 1000 live births. Furthermore, we confirmed if the residuals were normally distributed and whether or not the model exhibited homoscedasticity using residual plot and White’s test, respectively. In addition, Breusch-Pagan test was applied to confirm if the model exhibited homoscedasticity. Presence of multicollinearity was assessed using variance inflation factor and tolerance test. Statistical significance of the association was determined by two-tailed Wald test at significance level of alpha equal to 5%.

### Ethical approval

For analysis of anonymous publicly available data, no ethical approval is required.^21 23^

## Results

### Descriptive statistics

The summary statistics of the 49 countries included in the analysis are shown in table 1. Across countries neonatal mortality ranged from 8 to 50 per 1000 live births (average 30.1 per 1000 live births, SD 9.8 per 1000 live births). The prevalence of HIV among adults of reproductive age ranged from 0.2% to 26.5% (average 4.9%, SD 6.5%). The average literacy and poverty rates were 65% and 47%, respectively. Contraceptive use was reported for 28% of women; 59% of all deliveries were reported to be supervised by a skilled healthcare provider. An average of six health professionals cared for a population of 10 000. US$209.6 was spent on health per person per year with a wide disparity ranging from US$17 to US$1642 per person per year. Only eight countries (Botswana, Equatorial Guinea, Gabon, Mauritius, Seychelles, Swaziland, South Africa and Namibia) spent up to US$250 per person per year, the rest committed <US$100 per person per year. Quality of healthcare governance was low with an average of −4.1. Only the Seychelles, Mauritius, Cape Verde, Namibia, Botswana, Ghana and South Africa had a positive healthcare governance score.

**Table 1:** Descriptive characteristics of 49 sub-Saharan African countries

Country characteristics	Mean (SD; range)
Neonatal mortality (per 1000 live births)	30.1 (9.8; 8 to 50)
HIV prevalence (%)	4.9 (6.5; 0.2 to 26.5)
Health financing (US$ per person)	209.6 (313.4; 17 to 1642.7)
Health human resources (per 1000 people)	0.6 (0.9; 0.2 to 4.7)
Physicians per 1000 people	0.2 (0.28; 0.01 to 1.51)
*Nurses and midwives per 1000 people*	1.1 (1.46; 0.03 to 7.92)
Health service delivery (%)	43.4 (18.9; 1.8 to 87.6)
Contraceptive prevalence (%)	28.1 (19.3; 4 to 75.9)
Skilled delivery (%)	59.1 (21.9; 10 to 99.2)
Health governance (score ranged from −15 to +15)	- 4.1 (3.8; −13.7 to 5.0)
Country socioeconomic status	56.0 (14.9; 29.5 to 95.9)
Literacy rate (% of people aged 15 and above)	65.0 (19.1; 27 to 94.2)
Poverty rate (%)	47.5 (18.3; 2 to 76.8)
Access to improved water source (%)	70.6 (17.0; 29.5 to 99.8)
Access to improved sanitation facilities (%)	35.9 (23.0; 8.9 to 97.1)

### Special-cause and common-cause variations in neonatal mortality

**Figure 1:**
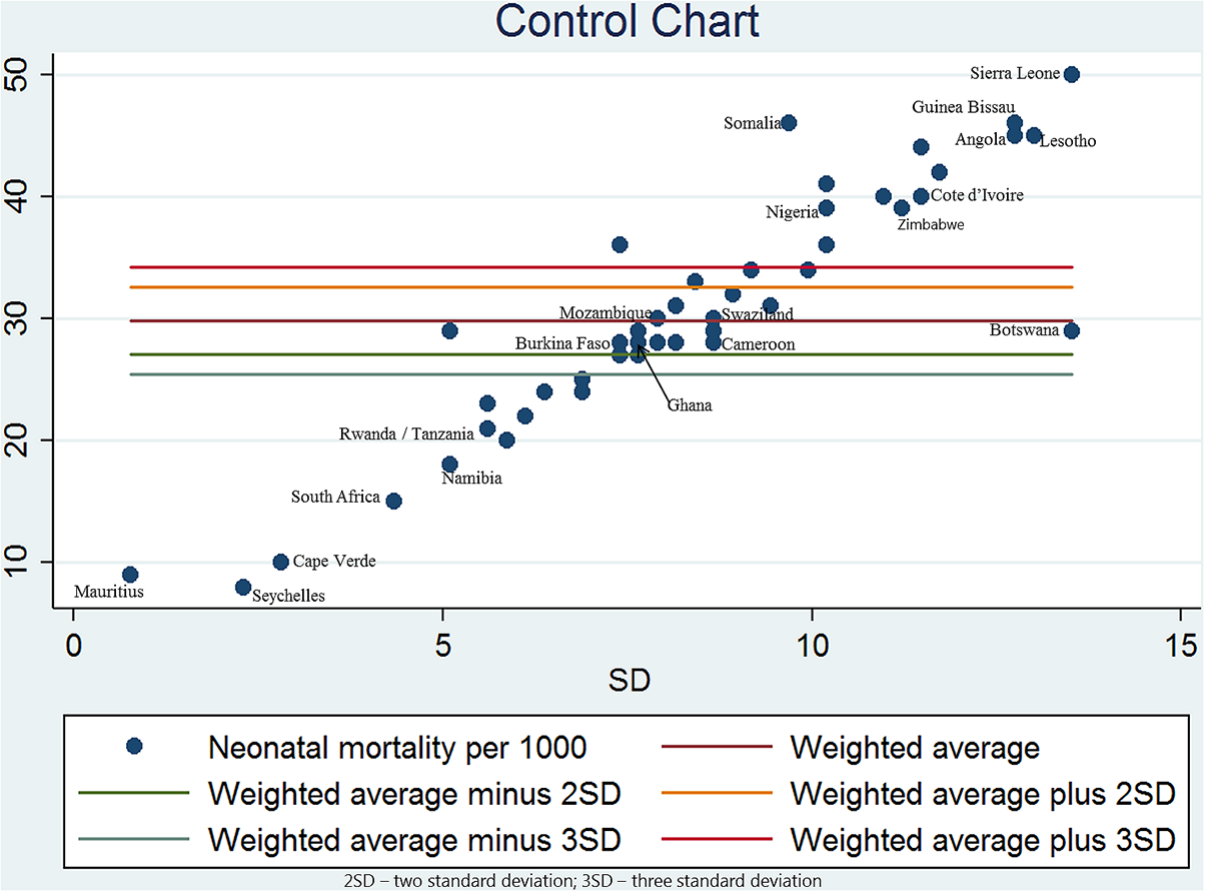
Control chart.

Figure 1 shows the results of the control chart that explored variation in neonatal mortality across 49 SSA countries. The weighted mean of neonatal mortality rates was 29.8 per 1000 live births. Neonatal mortality rates in 21 SSA countries (43%) were within the 99.8% control limits. Variation observed within this limits suggests common-cause variation. Neonatal mortality rates in 14 SSA countries were above and in 14 countries below the 99.8% control limits, indicating special-cause variation in 28 (57%) SSA countries.

### Geographical variation/clustering in neonatal mortality

Table 2 shows both global and local spatial autocorrelation for neonatal mortality among SSA countries. The global Moran’s I statistic (Moran’s I statistic=−0.0925; p=0.74) and Geary’s C statistic (Geary’s C statistic=1.0640; p=0.71) were not significant indicating absence of spatial clustering of neonatal mortality rates. The local spatial autocorrelation estimates for neonatal mortality were similarly not significant indicating absence of local spatial autocorrelation between any SSA country and neighbouring countries.

**Table 2:** Global and local spatial autocorrelation for neonatal mortality in sub-Saharan Africa

Country	Local Moran’s I statistic (p value)
Angola	−0.2034 (0.6473)
Benin Republic	−0.0077 (0.4859)
Botswana	0.2638 (0.2713)
Burkina Faso	−0.0706 (0.5488)
Burundi	−0.1874 (0.6139)
Cameroon	−0.1784 (0.6588)
Central Africa Republic	0.3505 (0.1849)
Chad	0.1301 (0.3559)
Côte d’Ivoire	−0.0065 (0.4829)
Congo Brazzaville	0.0095 (0.4677)
Congo Democratic Republic	−0.1832 (0.7052)
Djibouti	0.0103 (0.4750)
Equatorial Guinea	−0.1604 (0.5785)
Eritrea	0.4242 (0.2089)
Ethiopia	0.0662 (0.4142)
Gabon	0.0502 (0.4464)
Gambia	0.4236 (0.3243)
Ghana	−0.0975 (0.5525)
Guinea Liberia	0.1890 (0.2854)
Guinea-Bissau	−0.5647 (0.7843)
Kenya	0.1532 (0.3354)
Lesotho	−3.0964 (0.9991)
Liberia	−0.6433 (0.8681)
Malawi	0.5700 (0.1416)
Mali	−0.0743 (0.5527)
Mauritania	0.0342 (0.4660)
Mozambique	0.1434 (0.3279)
Namibia	0.0885 (0.3935)
Niger	−0.1910 (0.6549)
Nigeria	−0.0856 (0.5514)
Rwanda	0.1301 (0.3721)
Senegal	−0.5428 (0.8926)
Sierra Leone	−0.3441 (0.6792)
Somalia	−0.5647 (0.8354)
South Africa	0.0056 (0.4682)
Sudan	0.0057 (0.4651)
Swaziland	0.2429 (0.3486)
Tanzania	0.3956 (0.0925)
Uganda	−0.0616 (0.5268)
Togo	0.4280 (0.1396)
Zambia	0.0242 (0.4390)
Zimbabwe	−0.7644 (0.9617)

Global Moran’s I statistic=−0.0925; p=0.7393, Geary’s C statistic=1.0640; p=0.7116.

### Associations between country-level characteristic and neonatal mortality

**Figure 2:**
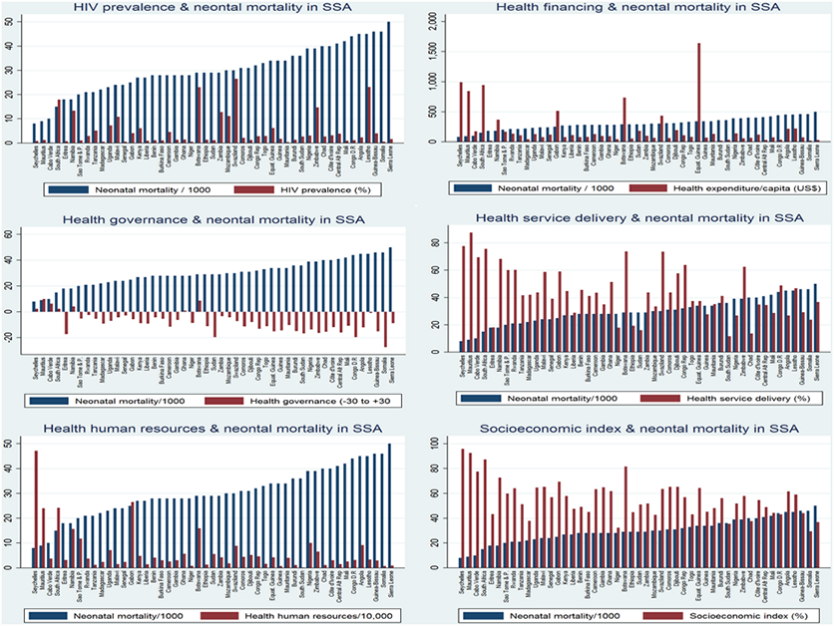
Relationships between country-level characteristics and neonatal mortality in sub-Saharan Africa (SSA).

The relationships between country-level characteristics (HIV prevalence, health financing, health human resources, health service delivery, health governance and country socioeconomic status) and neonatal mortality are shown in figure 2. No relationship was observed between HIV prevalence among the population of reproductive age and neonatal mortality. HIV prevalence rates were lowest for Cape Verde, Mauritania, Madagascar, Niger, Senegal and Somalia and highest for Swaziland, Lesotho, Botswana, South Africa, Zimbabwe and Namibia. In general, neonatal mortality was inversely related to healthcare financing, health human resource capacity, coverage of health service delivery quality of healthcare governance and socioeconomic index of the country.

**Figure 3:**
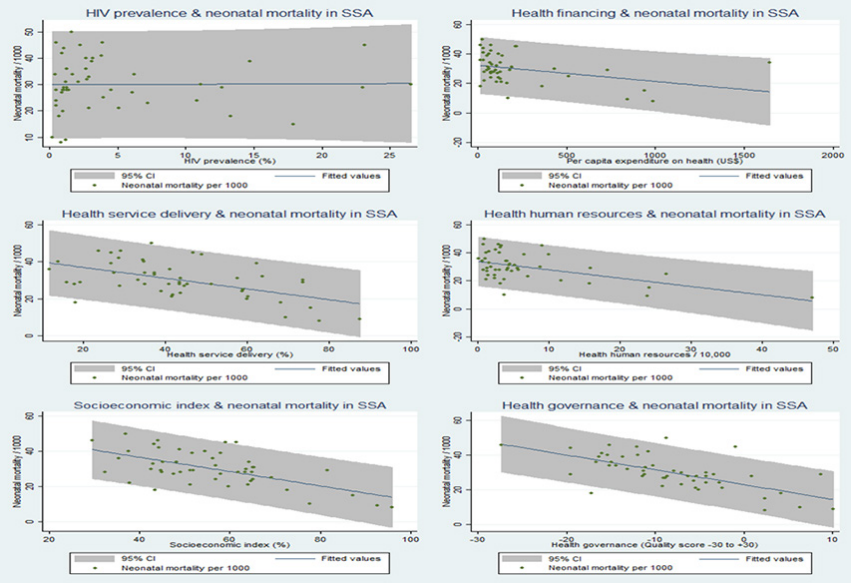
Unadjusted associations between country-level characteristics and neonatal mortality in sub-Saharan Africa (SSA).

**Figure 4:**
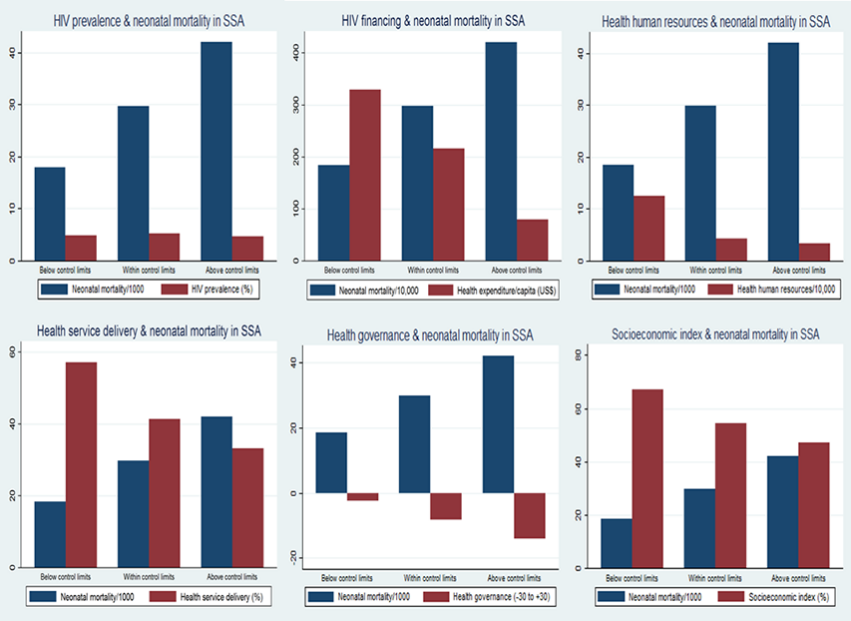
Relationships between country-level characteristics and neonatal mortality for countries below, within and above the control limits. SSA, sub-Saharan Africa.

Further quantitative assessment of relationships between country characteristics and neonatal mortality using two-way scatter plots with a prediction line and 95% CI showed no relationship between HIV prevalence and neonatal mortality (figure 3). Financial investment in health was inversely related to neonatal mortality (correlation coefficient r=0.35; p=0.01). Similarly, country’s health human resources capacity showed an inverse linear relationship with neonatal mortality (correlation coefficient r=0.52; p<0.001). An inverse relationship was observed between the population coverage of health service delivery and neonatal mortality (correlation coefficient r=0.54; p<0.001). As country’s quality of healthcare governance increased neonatal mortality decreased (correlation coefficient r=0.52; p<0.001); a similar relationship was observed for socioeconomic index (correlation coefficient r=0.61; p<0.001). The pooled characteristics (HIV prevalence, health financing, health human resources, health service delivery, quality of healthcare governance and country socioeconomic index) of the countries below, within and above the control limits (≈3 SD) were compared with average neonatal mortality using multiple double-bar charts as shown in figure 4. The results clearly show linear inverse relationships of the country’s health financing, health human resources, health service delivery coverage, health governance performance and socioeconomic index with neonatal mortality. HIV prevalence appeared not to have a significant relationship with neonatal mortality.

**Table 3:** Association between prespecified country characteristics and neonatal death

Country characteristics	Univariable model	Multivariable model
	β (95% CI)	β (95% CI)
HIV prevalence	0.019 (−0.429 to 0.467)	0.463 (0.135 to 0.790)**
Health financing	−0.011 (-0.020 to −0.002)*	###
Health human resources	−5.962 (-8.870 to −3.055)***	###
Health service delivery	−0.290 (-0.422 to −0.158)***	###
Health governance	−1.707 (-2.282 to −1.133)***	−1.327 (−2.073 to −0.580)**
Country socioeconomic index	−0.408 (-0.562 to −0.254)***	−0.234 (−0.424 to −0.044)*

*p<0.05; **p<0.01; ***p<0.001; β regression coefficient, ### variable omitted in the final model based on adjusted R^2^.

The results of the linear regression analysis to examine the association between country characteristic and neonatal death are shown in table 3. In the multivariable regression model, three country characteristics were observed to have a significant independent association with neonatal death. The quality of healthcare governance was the strongest underlying factor for neonatal survival; for every unit increase in country’s quality of healthcare governance neonatal mortality declined by −1.327 per 1000 live births (95% CI −2.073 to −0.580; p<0.01). In addition, for every unit decline in the percentage of population under socioeconomic deprivation, neonatal mortality declined by −0.234 per 1000 live births (95% CI −0.424 to −0.044; p<0.05), whereas for every unit increase in the prevalence of HIV/AIDS among the population of reproductive age, neonatal mortality increased by 0.463 per 1000 live births (95% CI 0.135 to 0.790; p<0.01).

## Discussion

This study shows a vast geographical variation in neonatal mortality across 49 SSA countries. A substantial part of this variation could be explained by the differences in quality of healthcare governance, prevalence of HIV among the population of reproductive age and socioeconomic index across SSA countries. Even though we applied the appropriate statistical methods for analysis and the data used can be perceived as reliable data considering their sources, our findings could be threatened by ecological fallacy; this seems, however, to be very unlikely for the observed relationship between the quality of healthcare governance and neonatal death. Residual confounding might have influenced the observed relationships as individual-level characteristics could not be considered. In addition, the temporal relationship between neonatal mortality and country characteristics could not be established in this type of study. Thus, it is impossible to make a causal inference. Our study showed that in this region, more than half of the residents are living in poverty, 1 out of 20 people is HIV-infected and the scarcity of human and financial resources makes it difficult to provide adequate health services and other basic necessities of life such as safe water and education. Our analyses do not show spatial clustering in neonatal mortality among SSA countries. This spatial randomness might be due to the use of data at country-level rather than at province or district level as employed in previous studies observing spatial clustering in childhood mortality and morbidity.^7 8^ The control chart analyses showed both common-cause and special-cause variation in neonatal mortality. Further examination of countries located below the control limits may identify factors that explain why these countries perform better than others. Similarly, countries above the control limits (>3 SD) should be thoroughly assessed to identify factors responsible for their excess incidence of neonatal mortality. While countries within the 99.8% control limits show no evidence of special-cause variation, it remains to be explained why they have higher neonatal mortality rate than those below the control limits.

Globally, the leading direct causes of neonatal deaths are: prematurity/low birth weight, birth asphyxia, neonatal sepsis and birth trauma.^1^ Indirect factors also contribute to neonatal death by influencing neonatal survival at both individual level and population level.^30^ The present study investigated the associations between neonatal mortality and indirect factors by identifying differences in country characteristics that might be responsible for the observed variations in neonatal mortality in SSA. Quality of healthcare governance was the strongest determinant of neonatal mortality in this study, although only 7 of the 49 SSA countries showed a positive rating in the quality of healthcare governance. To date, no previous studies in the literature have linked the quality of healthcare governance with neonatal mortality. Generally, emphasis has been placed on increasing health financing and health service coverage which is commendable, but only in the presence of good healthcare governance can this be fully effective. Without ensuring good quality in healthcare governance, scarce healthcare resources will not be used judiciously as shown by the results of a diagnostic public expenditure tracking survey conducted in Ghana, Uganda and Tanzania that demonstrated extensive leakages of public funds in these three SSA countries.^31^ Thus, genuine commitment by governments is required to improve the quality of healthcare governance by ensuring transparency and accountability, government effectiveness, political stability, adherence to the rule of law, high regulatory quality and stiffer disciplinary action against corruption.

HIV prevalence among the population of reproductive age was positively associated with neonatal death, consistent with findings from a previous study that showed that neonates of HIV-positive mothers were more likely to die.^32^ This is yet another argument to promote prevention of mother-to-child transmission. Neonatal mortality declined as country’s socioeconomic index improved, a result in line with previous studies observing that dwelling in a socioeconomically deprived population increases the risk of childhood morbidity and mortality.^30 33–35^ Similarly, a multicountry study that involved 13 SSA countries showed that the likelihood of dying at neonatal stage among the families in the poorest quintile was almost 70% higher than in the richest quintile.^36^ Implementing free basic education and poverty alleviation programme in conjunction with expansion of safe water supply and promotion of effective sanitation programme will help to improve neonatal survival. In conclusion, the results of this study based on data from 49 SSA countries show a marked variation in neonatal mortality. A substantial part of this variation can be explained by differences in health governance performance, prevalence of HIV and socioeconomic deprivation.
